# Symmetry of molecular Rydberg states revealed by XUV transient absorption spectroscopy

**DOI:** 10.1038/s41467-019-13251-w

**Published:** 2019-11-21

**Authors:** Peng Peng, Claude Marceau, Marius Hervé, P. B. Corkum, A. Yu. Naumov, D. M. Villeneuve

**Affiliations:** Joint Attosecond Science Laboratory, National Research Council and University of Ottawa, Ottawa, ON K1A 0R6 Canada

**Keywords:** Atomic and molecular interactions with photons, Attosecond science

## Abstract

Transient absorption spectroscopy is utilized extensively for measurements of bound- and quasibound-state dynamics of atoms and molecules. The extension of this technique into the extreme ultraviolet (XUV) region with attosecond pulses has the potential to attain unprecedented time resolution. Here we apply this technique to aligned-in-space molecules. The XUV pulses are much shorter than the time during which the molecules remain aligned, typically $<$100 fs. However, transient absorption is not an instantaneous probe, because long-lived coherences re-emit for picoseconds to nanoseconds. Due to dephasing of the rotational wavepacket, it is not clear if these coherences will be evident in the absorption spectrum, and whether the properties of the initial excitations will be preserved. We studied Rydberg states of N${}_{2}$ and O${}_{2}$ from 12 to 23 eV. We were able to determine the polarization direction of the electronic transitions, and hence identify the symmetry of the final states.

## Abstract (web summary)

Transient absorption spectroscopy is used to identify the structural characteristics of the atoms and molecules. Here the authors used extreme ultraviolet transient absorption spectroscopy to identify the Rydberg state symmetry of aligned molecules.

## Introduction

XUV transient absorption spectroscopy uses coherent extreme ultraviolet (XUV) pulses to probe the electronic structure of atoms and molecules. Attosecond transient absorption spectroscopy (ATAS) uses high harmonic generation to create a broad spectrum in the XUV or soft X-ray range. The broad spectrum permits the recording of many absorption lines in parallel^1–17^. ATAS can probe valence electrons through excitation to high-lying electronic states^1–14^. ATAS can also excite core-level electrons to reveal molecular structure using techniques such as near-edge X-ray absorption fine structure (NEXAFS)^15–18^, for example around the carbon K-edge at 284 eV. Pump-probe type experiments in which two-photon processes are driven by a combination of an attosecond pulse and an infrared pulse have revealed coupling between states and the production of light-induced states^4,7^. Shifts in absorption features have been used to observe vibrational motion in bromine molecules^14^.

When linear molecules are irradiated by a infrared pump pulse whose duration is much shorter than the rotational period, nonadiabatic field-free alignment is achieved^19–21^. Each initial thermally-populated rotational eigenstate $\left|{J}_{0},{M}_{0}\right\rangle$ will expand to a rotational wave packet.

1 $${\Psi }_{{J}_{0},{M}_{0}}(t)=\sum _{J,M}{a}_{J,M}^{{J}_{0},{M}_{0}}(t)\left|J,M\right\rangle ,$$

where $J$ is the rotational quantum number, $M$ is the projection on the polarization axis and ${a}_{J,M}^{{J}_{0},{M}_{0}}(t)$ is the amplitude for each $\left|J,M\right\rangle$ state. The time evolution of the wave packet can be calculated by solving the time-dependent Schrodinger equation. The degree of alignment is characterized by:

2 $${\langle {\cos }^{2}\theta \rangle }_{{J}_{0},{M}_{0}}(t)=\langle {\Psi }_{{J}_{0},{M}_{0}}(t)| {\cos }^{2}\theta | {\Psi }_{{J}_{0},{M}_{0}}(t)\rangle ,$$

in which $\theta$ is the angle of the molecular axis to the polarization axis. Finally, thermally averaged values of the degree of alignment are obtained as:

3 $$\langle {\cos }^{2}\theta \rangle (t)=\sum _{{J}_{0},{M}_{0}}{\rho }_{{J}_{0},{M}_{0}}(T){\langle {\cos }^{2}\theta \rangle }_{{J}_{0},{M}_{0}}(t),$$

where $T$ is the rotational temperature and ${\rho }_{{J}_{0},{M}_{0}}(T)$ is the Boltzmann weight function for the $\left|{J}_{0},{M}_{0}\right\rangle$ initial states. $\langle {\cos }^{2}\theta \rangle (t)$ varies with time after the aligning laser pulse as various rotational states go in and out of phase. The probe attosecond pulses arrive at a revival of the rotational wavepacket at which the value of $\langle {\cos }^{2}\theta \rangle$ maximizes. The electronic excitation is in the molecular frame, but observations occur in the laboratory frame; the frames are related through the Wigner rotation matrix. The free induction decay resulting from the induced polarization in the molecule frame continues to radiate while the rotational wavepacket dephases. At times the radiation associated with different rotational eigenstates will be out of phase and will lead to cancellation of the emission. In addition, the rotational constants of the lower and upper states will be different. Therefore it needs to be demonstrated that the time-integrated absorption of aligned molecules will exhibit the characteristics of the attosecond excitation.

In the following, we use XUV transient absorption spectroscopy to record the molecular absorption spectra of N${}_{2}$ and O${}_{2}$. The short pulse duration of attosecond pulse trains (typically 5–30 fs, but as short as 50 as refs. ^22,23^) permits using a near-infrared (NIR) laser pulse to impulsively align the molecular sample^19–21^, allowing the absorption measurements to be made in a sample in which the molecular axes can be aligned relative to the polarization axis of the probing XUV field. This is not possible in synchrotron experiments due to their relatively long pulse durations. We will show that we can discriminate between parallel and perpendicular transitions for linear molecules, and hence identify the symmetry of the final states. In the case of O${}_{2}$, the observed symmetry of some states differs from previous assignments. This same approach can be applied to larger molecules, for example asymmetric top molecules^24^.

## Results

### XUV transient absorption in N${}_{2}$ and O${}_{2}$

**Fig. 1:**
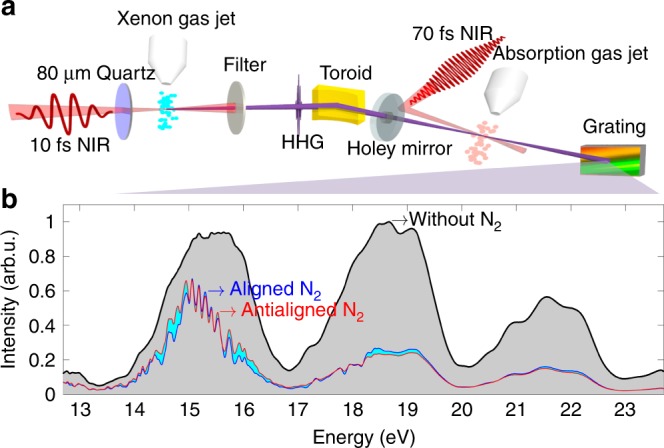
Experimental setup and XUV spectra. **a** Schematic of the experimental setup. A broad XUV spectrum is generated by focusing a 10 fs NIR pulse into a xenon gas jet to generate an attosecond pulse train. The XUV is refocused in a second gas jet containing the target gas. A longer NIR pulse is used to impulsively align the molecules in the second gas jet. **b** XUV spectra taken without the target gas (black curve), after absorption by N${}_{2}$ at the delay times corresponding to alignment (blue curve) and anti-alignment (red curve).

 Figure 1a illustrates the experimental arrangement. A 10 fs NIR pulse centered at 800 nm was focused into a pulsed gas jet of xenon for high harmonic generation (see Methods for more details). The bandwidth of each harmonic was 2.3 eV and the total spectrum spanned 12–24 eV, as shown by the black curve in Fig. 1b. A 70 fs NIR pulse was used to perform impulsive alignment on the sample molecules. It was combined with the XUV pulse with a holey mirror. The alignment pulse was displaced from the center of the holey mirror while the XUV pulse passed through the hole. Both the XUV and the alignment pulses were vertically polarized, they propagated noncollinearly and intersected at the absorption gas jet with a crossing angle of 18 mrad. The transmitted XUV spectra were recorded at different time delays $\tau$ between the alignment and XUV pulses. The blue (red) curve in Fig. 1(b) shows the transmitted XUV spectrum at aligned delay $\tau =8.57\ {\rm{ps}}$ (anti-aligned delay $\tau =8.32\ {\rm{ps}}$) for N${}_{2}$. About 10,000 laser shots were accumulated at each delay point.

**Fig. 2:**
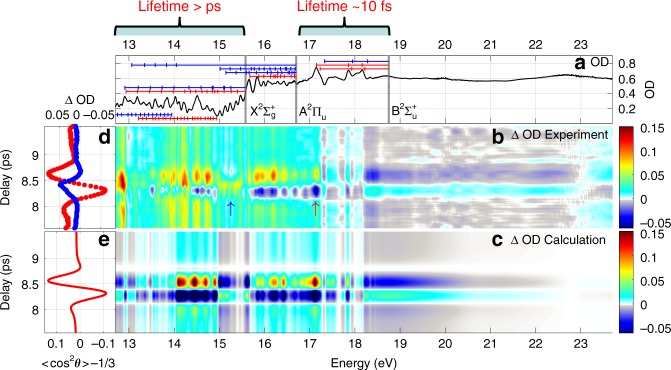
Experimental and calculated XUV transient absorption in aligned N${}_{2}$. **a** Static absorption of isotropic N${}_{2}$ molecules from 12.7 to 23.7 eV. The positions of vibrational levels of the valence ${b}^{1}{\Pi }_{u}$ and *b*’${}^{1}{\Sigma }_{u}^{+}$ states and of the Rydberg series built on ${{\rm{X}}}^{2}{\Sigma }_{{\rm{g}}}^{+},{{\rm{A}}}^{2}{\Pi }_{{\rm{u}}}$ and ${{\rm{B}}}^{2}{\Sigma }_{{\rm{u}}}^{+}$ N${}_{2}^{+}$ cores are indicated, parallel and perpendicular transitions are labeled by red and blue lines respectively. **b** Experimental and **c** calculated differential absorption spectra around the full revival of aligned N${}_{2}$ molecules. **d** Modulations of the spectrum at 15.28 eV (blue circle) and 17.14 eV (red circle). **e** Calculated alignment degree $\langle {\cos }^{2}\theta \rangle -1/3$.

We define the differential absorption spectrum to be the differential optical density $\Delta {\rm{OD}}(E,\tau )=-{\mathrm{log}}_{10}[{I}_{{\rm{on}}}(E,\tau )/{I}_{{\rm{off}}}(E,\tau )]$. Here, ${I}_{{\rm{on}}}(E,\tau )$ and ${I}_{{\rm{off}}}(E,\tau )$ represent the XUV spectra after transmission through the sample, taken with and without the alignment field, respectively. This is effectively the XUV absorption of the aligned medium minus that of the randomly aligned medium Fig. 2a shows the static absorption of isotropic N${}_{2}$ measured by blocking the NIR alignment pulse. The ground state configuration may be written as ${(1{\sigma }_{{\rm{g}}})}^{2}{(1{\sigma }_{{\rm{u}}})}^{2}{(2{\sigma }_{{\rm{g}}})}^{2}{(2{\sigma }_{{\rm{u}}})}^{2}{(1{\pi }_{{\rm{u}}})}^{4}{(3{\sigma }_{{\rm{g}}})}^{2} \, \, {{\rm{X}}}^{1}{\Sigma }_{{\rm{g}}}^{+}$. The adiabatic ionization energies corresponding to different final states of N${}_{2}^{+}$ are ${(3{\sigma }_{{\rm{g}}})}^{-1}{{\rm{X}}}^{2} \, \, \,{\Sigma }_{{\rm{g}}}^{+}=15.58\ {\rm{eV}}$, ${(1{\pi }_{{\rm{u}}})}^{-1}{{\rm{A}}}^{2} \, \, {\Pi }_{{\rm{u}}}=16.70\ {\rm{eV}}$, ${(2{\sigma }_{{\rm{u}}})}^{-1}{{\rm{B}}}^{2} \, \, {\Sigma }_{{\rm{u}}}^{+}=18.75\ {\rm{eV}}$^25–27^, as shown by the thick gray lines in Fig. 2a. The XUV pulse excites numerous overlapping vibrational series associated with states of Rydberg and valence character. The lifetimes of these states are quite different. The states between 17 and 18.5 eV have short lifetimes of $\sim$10 fs due to autoionization^12^ and the absorption peaks show wide asymmetric Fano line shapes, whereas states below the ionization potential (15.58 eV) have long lifetime $> {\rm{ps}}$^28,29^ and the absorption peaks show narrow symmetric Lorentzian line shapes. Following the assignment of^30–33^, the absorption features are summarized in Table 1. We use red (blue) lines to label the excited states with ${}^{1}{\Sigma }_{{\rm{u}}}^{+}$ (${}^{1}{\Pi }_{{\rm{u}}}$) symmetry. Due to the finite energy resolution of the XUV spectrometer (70 meV), the full structure of the excited states cannot be resolved. Figure 2b shows the differential absorption spectrum measured around the full rotational revival delay of N${}_{2}$. The alignment-induced absorbance modulation can be seen across the whole energy range, which shows that both long-lived states and short-lived autoionizing states are sensitive to molecular alignment at the time of the probe signal. Figure 2d shows lineouts versus time delay for the absorption peaks at 15.28 eV (marked by the blue arrow) and 17.14 eV (marked by the red arrow); the different modulation depth and polarity are evident.

**Table 1:** Assignment of ${{\rm{N}}}_{2}$ absorption spectrum.

$\epsilon$ (eV)	Transition	Description	${{\rm{N}}}_{2}^{+}{\rm{core}}$
12.7–15.58	$(1{\pi }_{u})(3{\sigma }_{g})\to {(1{\pi }_{g})}^{2}$	${{\rm{b}}}^{1}{{\rm{\Pi}} }_{{\rm{u}}}^{{\rm{a}}}$	Valence
	$$2{\sigma }_{u}\to 1{\pi }_{g}$$		
	$3{\sigma }_{g}\to 3{\sigma }_{u}$	b’${}^{1}{{\rm{\Sigma}} }_{{\rm{u}}}^{{+}^{{\rm{a}}}}$	Valence
	$$1{\pi }_{u}\to 1{\pi }_{g}$$		
	$3{\sigma }_{g}\to np{\sigma }_{u}$	CY${}^{{\rm{b}}}$	${{\rm{X}}}^{2}{{\rm{\Sigma}} }_{{\rm{g}}}^{+}$
	$3{\sigma }_{g}\to np{\pi }_{u}$	WJ${}^{{\rm{b}}}$	
	$1{\pi }_{u}\to 3s{\sigma }_{g}$	${{\rm{o}}}_{3}^{{\rm{a}}}$	${{\rm{A}}}^{2}{{\rm{\Pi}} }_{{\rm{u}}}$
15.58–16.70	$1{\pi }_{u}\to nd{\delta }_{g}$	WT${}^{{\rm{b}}}$	
	$1{\pi }_{u}\to nd{\sigma }_{g}$	OT${}^{{\rm{b}}}$	
	Unassigned	UN${}^{{\rm{c}},{\rm{d}}}$	
16.70–18.75	$2{\sigma }_{u}\to nd{\sigma }_{g}$	HA${}^{{\rm{b}},{\rm{c}}}$	${{\rm{B}}}^{2}{{\rm{\Sigma}} }_{{\rm{u}}}^{+}$
	$2{\sigma }_{u}\to nd{\pi }_{g}$	HE${}^{{\rm{b}},{\rm{c}}}$	
	$2{\sigma }_{u}\to ns{\sigma }_{g}$	New OT${}^{{\rm{b}},{\rm{c}}}$	

The experimental absorption spectrum of N_2_, shown in Fig. 2a, consists of numerous overlapping vibrational series associated with states of Rydberg and valence character. The absorption features are summarized by the photon energy $\epsilon$, the column labeled “Description" is the name of the electronic transitions in the “Transition" column, the “N_2_^+^ core" column is the ionic state, to which the Rydberg series converge

*CY* Carroll Yoshino, *WJ* Worley Jenkins, *WT* Worley third, *OT* Ogawa Tanaka, *UN* Unassigned, *HA* Hopfield absorption, *HE* Hopfield emission

^a^Reference^30^

^b^Reference^31^

^c^Reference^32^

^d^Reference^33^

Figure 2b clearly shows that there are two types of absorption features: those that maximize when the molecular axis is parallel to the XUV polarization direction ($\tau =8.57\ {\rm{ps}}$), and those that maximize when the molecular axis is perpendicular ($\tau =8.32\ {\rm{ps}}$). For example, at 15 eV, the direction changes, as it does at 18.2 eV. Visually one can identify regions of parallel or perpendicular electronic transitions, illustrating the power of ATAS with aligned molecules.

**Fig. 3:**
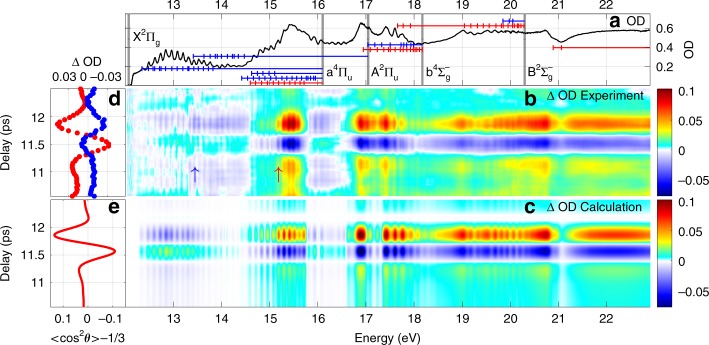
Experimental and calculated XUV transient absorption in aligned O${}_{2}$. **a** Static absorption of isotropic O${}_{2}$ molecules from 12 to 22.9 eV. The positions of vibrational levels of Rydberg series built on several O${}_{2}^{+}$ cores are indicated, parallel and perpendicular transition are labeled by red and blue lines respectively. **b** Experimental and **c** calculated differential absorption spectra around the full revival of aligned O${}_{2}$ molecules. **d** Modulations of the spectrum at 13.43 eV (blue circle) and 15.18 eV (red circle). **e** Calculated alignment degree $<{\cos }^{2}\theta > -1/3$.

We now turn to O${}_{2}$. Figure 3a shows the static absorption of isotropic O${}_{2}$. The ground state may be written as ${(1{\sigma }_{{\rm{g}}})}^{2}{(1{\sigma }_{{\rm{u}}})}^{2}{(2{\sigma }_{{\rm{g}}})}^{2}{(2{\sigma }_{{\rm{u}}})}^{2}{(3{\sigma }_{{\rm{g}}})}^{2}{(1{\pi }_{{\rm{u}}})}^{4}{(1{\pi }_{{\rm{g}}})}^{2} \, \,{{\rm{X}}}^{3}{\Sigma }_{{\rm{g}}}^{-}$. The adiabatic ionization energies corresponding to different final states of O${}_{2}^{+}$ are ${(1{\pi }_{{\rm{g}}})}^{-1} \, \, {{\rm{X}}}^{2}{\Pi }_{{\rm{g}}}=12.07\ {\rm{eV}}$, ${(1{\pi }_{{\rm{u}}})}^{-1} \, \, {{\rm{a}}}^{4}{\Pi }_{{\rm{u}}}=16.1\ {\rm{eV}}$, ${(1{\pi }_{{\rm{u}}})}^{-1} {{\rm{A}}}^{2}{\Pi }_{{\rm{u}}}=17.05\ {\rm{eV}}$, ${(3{\sigma }_{{\rm{g}}})}^{-1} \,\, {{\rm{b}}}^{4}{\Sigma }_{{\rm{g}}}^{-}=18.17\ {\rm{eV}}$, ${(3{\sigma }_{{\rm{g}}})}^{-1} \, \, {{\rm{B}}}^{2}{\Sigma }_{{\rm{g}}}^{-}=20.3\ {\rm{eV}}$, ${(2{\sigma }_{{\rm{u}}})}^{-1} \, \, {{\rm{c}}}^{4}{\Sigma }_{{\rm{u}}}^{-}=24.56\ {\rm{eV}}$^34,37^, as shown by the thick gray lines in Fig. 3(a). Following the assignment of^34–36^, the absorption features are summarized in Table 2. Similarly, we use red (blue) lines to label the excited states with ${}^{3}{\Sigma }_{{\rm{u}}}^{-}$ (${}^{3}{\Pi }_{{\rm{u}}}$) symmetry. Figure 3b shows the differential absorption spectra and Fig. 3d shows lineouts for the absorption peak at 13.43 eV (marked by blue arrow) and 15.18 eV (marked by red arrow).

**Table 2:** Assignment of ${{\rm{O}}}_{2}$ absorption spectrum.

$\epsilon$ (eV)	Transition	Description	${{\rm{O}}}_{2}^{+}{\rm{core}}$
12.3–17.05	$1{\pi }_{{\rm{u}}}\to 3{\rm{s}}{\sigma }_{{\rm{g}}}$	${{\rm{H}}}^{{\rm{a}}}$	${{\rm{a}}}^{4}{{\rm{\Pi}} }_{{\rm{u}}}$
	$1{\pi }_{{\rm{u}}}\to 4{\rm{s}}{\sigma }_{{\rm{g}}}$	${{\rm{I}}}^{{\rm{b}},{\rm{c}}}$	
	$1{\pi }_{{\rm{u}}}\to 3{\rm{d}}{\delta }_{{\rm{g}}}$	I’${}^{{\rm{b}},{\rm{c}}}$	
	$1{\pi }_{{\rm{u}}}\to 3{\rm{d}}{\sigma }_{{\rm{g}}}$	I”${}^{{\rm{b}},{\rm{c}}}$	
	$1{\pi }_{{\rm{u}}}\to 3{\rm{s}}{\sigma }_{{\rm{g}}}$	${{\rm{J}}}^{{\rm{a}}}$	${{\rm{A}}}^{2}{{\rm{\Pi}} }_{{\rm{u}}}$
17.05–20.3	$3{\sigma }_{g}\to np{\sigma }_{u}$	Strong${}^{{\rm{a}}}$	${{\rm{b}}}^{4}{{\rm{\Sigma}} }_{{\rm{g}}}^{-}$
	$3{\sigma }_{g}\to np{\pi }_{u}$	Weak${}^{{\rm{a}}}$	
	$3{\sigma }_{g}\to np{\sigma }_{u}$	Strong${}^{{\rm{a}}}$	${{\rm{B}}}^{2}{{\rm{\Sigma}} }_{{\rm{g}}}^{-}$
	$3{\sigma }_{g}\to np{\pi }_{u}$	Weak${}^{{\rm{a}}}$	
20.3–21.5	$2{\sigma }_{u}\to 3s{\sigma }_{g}$	Strong${}^{{\rm{a}}}$	${{\rm{c}}}^{4}{{\rm{\Sigma}} }_{{\rm{u}}}^{-}$

The experimental absorption spectrum of O_2_, shown in Fig. 3a, consists of numerous overlapping vibrational series associated with states of Rydberg and valence character. The absorption features are summarized by the photon energy $\epsilon$, the column labeled “Description" is the name of the electronic transitions in the “Transition" column, the “O_2_^+^ core" column is the ionic state, to which the Rydberg series converge

^a^Reference^34^

^b^Reference^35^

^c^Reference^36^

The experimental results in Figs. 2 and 3 show that the transient absorption signal indeed depends on the orientation of the molecules at the time of the XUV pulse, answering the question that we posed in the introduction. But it is not clear why this is true. The pump pulse creates a coherence between rotational states in the electronic ground state. At a later time the XUV pulse coherently excites the ensemble of molecules to a number of excited electronic states. The superposition of states results in an induced time-dependent polarization which will radiate an electric field for many picoseconds. Because the emission is out of phase with the XUV pulse, the time-integrated emission will appear as absorption^5,9^. For an atom, the emission will decay exponentially over picoseconds or nanoseconds due to the lifetime of the excited state, and thereby determines the spectral linewidth of the absorption feature. For molecules, the emitted polarization is more complicated, because the molecules continue to rotate after the XUV pulse.

### Calculation of induced polarization

**Fig. 4:**
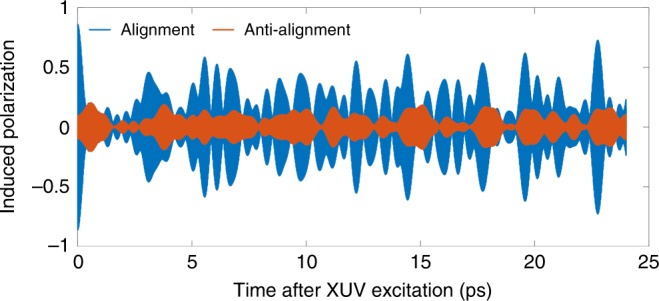
Calculated induced dipole moment for a rigid rotor linear molecule based on N${}_{2}$. A rotational wavepacket is created in the ground state by the pump pulse, and the XUV pulse arrives at a later time ${t}_{x}$. If ${t}_{x}$ is during a rotational revival (blue), then a greater polarization is induced. On the other hand, if ${t}_{x}$ is when the molecules are perpendicular to the polarization, then fewer molecules are excited. A larger induced polarization signal causes a greater absorption in the frequency spectrum. The rotational constants are ${B}_{0}=2$ cm${}^{-1}$ and ${B}_{1}=1.9$ cm${}^{-1}$.

To understand the time-domain picture of re-emission, we illustrate the time-varying polarization for a simplified rigid rotor modeled on the N${}_{2}$ molecule. We consider rotational states with $J$ in the range 0–10. We start with only $J=0,M=0$ and create a rotational wavepacket in the ground electronic state $\left|0\right\rangle$ by coherently populating the even $J$s via Raman transitions. This determines the set of ${a}_{J}$. The rotational wavepacket has a full revival at $t=8.5$ ps, just after an anti-revival at $t=8.2$ ps. At a delayed time ${t}_{x}$, the XUV pulse creates population in an excited electronic state $\left|1\right\rangle$ through dipole transitions. We consider a parallel transition, i.e. one which is allowed when the XUV polarization is parallel to the molecular axis. Rotational selection rules require that $\Delta J=\pm \!1$, so a set of odd-$J$ rotational states are populated with complex amplitudes ${b}_{J}$, creating a second rotational wavepacket in the excited electronic state. The induced polarization is proportional to the time-dependent dipole moment of the molecule.

4 $$\begin{array}{ccc}\mathrm{d}(t)&=&\langle {\psi }^{1}(t)| r| {\psi }^{0}(t)\rangle +c.c.\hfill\\ &=&\sum \limits_{JJ^{\prime} }{a}_{J}{b}_{J^{\prime} }^{* }{e}^{i{E}_{J^{\prime} }^{1}(t-{t}_{x})}{e}^{-i{E}_{J}^{0}t}\ {d}_{| | }\langle J^{\prime} M| \cos \theta | JM\rangle +c.c.,\end{array}$$

here ${E}_{J}^{0}$ and ${E}_{J'}^{1}$ are the energies of the eigenstates, including both rotational and electronic parts, ${d}_{| | }$ is the parallel component of the electronic transition dipole moment in the molecular frame. ${\mathrm{d}}(t)$ will have complex behavior in time, especially if the two electronic states have different rotational constants. See the Supplementary Note 1 for more details. Figure 4 shows a calculation of the induced polarization for this simplified molecule. The polarization results in free induction decay from the excited states. The re-emission is out of phase with the incident attosecond pulse, resulting in absorption lines in the transmitted spectrum^5,9,10^. A stronger polarization signal is associated with stronger absorption. The high frequency oscillations are mainly determined by the electronic transition frequency (14 eV in this calculation, not visible on this time scale). The low frequency oscillations are induced by the coherence between different rotational states and different rotational rates between the ground and excited states. This dipole leads to an absorption structure around 14 eV, which consists of several peaks, the separations of which are related to the rotational energy ($\sim$meV, which cannot be resolved by our XUV spectrometer). Probe times ${t}_{x}$ at the rotational revival (blue) and the anti-revival (red) are shown. The orientation-dependent behavior is evident during the first picosecond. For aligned molecules, the polarization signal is greatest immediately after excitation. For anti-aligned molecules, the signal is at first small, then increases as the molecules move into alignment 300 fs later. The overall signal is greater when the probe pulse arrives during the molecular alignment. This agrees with our experimental result: parallel transition shows stronger absorption when the XUV pulse arrives during a time of maximum alignment to the polarization direction.

One can understand this behavior classically. If the probe pulse arrives during a rotational revival, then more excited state population is produced for a parallel transition, and the excited molecules will also be parallel to the polarization direction. But the molecules in the ground and excited states will rotate at different rates, meaning that the induced dipole will modulate at their difference frequency. This leads to the modulations seen in Fig. 4.

### Frequency domain model

We now proceed to model the expected absorption in the frequency domain. More details are provided in the Supplementary Note 2. Impulsive alignment of a rigid rotor results in a distribution of molecular angles $\theta$ relative to the polarization axis $\hat{z}$ of the laser pulse. The distribution is cylindrically symmetric about $\hat{z}$ and varies in time based on the rotational constant of the molecule and its nuclear spin statistics^19^. The degree of alignment is characterized by the expectation value of the cosine-squared of the angle of the molecular axes to $\hat{z}$, namely $\langle {\cos }^{2}\theta \rangle (t)$. For an isotropic sample, $\langle {\cos }^{2}\theta \rangle =1/3$; for a sample perfectly aligned along direction $\hat{z}$ it is 1. At characteristic times called rotational revivals, the molecular axis distribution achieves its maximum degree of alignment. Just before a full revival, the molecular axes lie mostly in a plane perpendicular to $\hat{z}$, call anti-alignment, quickly followed by a maximum degree of alignment parallel to $\hat{z}$. Figures 2e and 3e show the calculated evolution of $\langle {\cos }^{2}\theta \rangle (t)-1/3$ in the region of the rotational revival.

For molecules of ${D}_{\infty h}$ symmetry group, all bound-bound electronic transitions must be either parallel or perpendicular to the molecular axis^38^. The measured absorption will then be a mixture of both types, weighted by the degree of alignment,

5 $$\sigma (E,t)=\langle {\cos }^{2}\theta \rangle (t)\cdot {\sigma }_{| | }(E)+\left[1-\langle {\cos }^{2}\theta \rangle (t)\right]\cdot {\sigma }_{\perp }(E),$$

where ${\sigma }_{| | }(E)$ and ${\sigma }_{\perp }(E)$ are absorption cross sections for parallel and perpendicular transitions. The absorption cross section for bound-bound transitions are taken from the high-resolution spectra of ref. ^25^ for N${}_{2}$ and ref. ^34^ for O${}_{2}$, including their symmetry assignments (summarized in Tables 1 and 2). The absorption cross sections for bound-free transitions above each ionization threshold are assumed to be flat in energy and are fit to the experiment, as described in the Supplementary Note 2. The calculated transmitted spectra are convolved with the experimental instrumental response function of 70 meV width, and the predicted differential absorption spectrum $\Delta {\rm{OD}}$ is calculated in the same way as for the experimental data. The model results are shown in Fig. 2c for N${}_{2}$ and Fig. 3c for O${}_{2}$.

The experimental and the calculated results are in good agreement. The significant difference around 13 eV for N${}_{2}$ may come from several factors. First, the XUV signal itself is weak around 13 eV as shown in Fig. 1(b). Second, the absorption cross section is huge ($\sim$400 Mb) as shown in the Supplementary Fig. 3, which means the XUV spectrum after absorption is very weak and the signal-to-noise ratio is poor in this energy range. Third, the second order diffraction of higher energy XUV photons (26 eV) falls onto 13 eV, which may pollute the signal. The simulated signal contrast is also larger than the measured contrast. One possible explanation is the assumption of full coherence of the rotational wavepacket in the ground state, by the use of Schrodinger’s equation. A more accurate simulation would instead use a density matrix approach in which a degree of incoherence in the rotational wavepacket is included. Another possible reason is the assumption of a constant spectrometer resolution in the whole spectrum range in the simulation, whereas the real resolution changes with photon energy (better resolution at lower photon energy).

## Discussion

The photoabsorption spectra of small molecules have been extensively studied using high-resolution XUV monochromators at synchrotron radiation facilities^25,34,39–42^. Large numbers of observed bands in the absorption spectra have been grouped into Rydberg series converging to different vibrational levels of various ion states. But one cannot get information on symmetries of photoexcited states from traditional photoabsorption spectroscopy without the help of theoretical calculations^43^. The Rydberg orbitals are usually determined by calculating the effective quantum number n* and quantum defect $\delta$, then the symmetry of the Rydberg state can be classified according to the build-up principles given by Herzberg^44^. Normally, assignments can be confirmed from the rotational analysis of the Rydberg transition or from the analysis of fluorescence polarization. However, few such data are available. In O${}_{2}$, the lifetime broadening due to rapid autoionization and/or predissociation results in the absence of clearly resolved rotational structure^34,45^. So the symmetry assigned to some Rydberg series are tentative and various interpretations exist^35,36,46–50^.

**Fig. 5:**
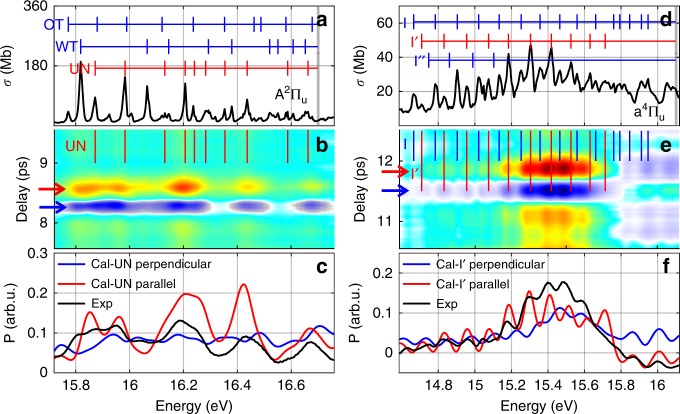
Assigning symmetry to some Rydberg series of N${}_{2}$ and O${}_{2}$. **a** High resolution absorption spectrum of N${}_{2}$ adapted from^25^, UN refers to unassigned states. **b** Zoom-in of Fig. 2b. **c** Measured and calculated asymmetry signals. **d** High resolution absorption spectrum of O${}_{2}$ adapted from ref. ^34^. **e** Magnification of Fig. 3b. **f** Measured and calculated asymmetry signals.

Despite extensive experimental and theoretical work on the assignments of the Rydberg series converging onto the N${}_{2}^{+}$ X, A, and B states, a consistent interpretation has yet to emerge^46^. The main difference between these assignments concerns the Rydberg series converging onto the N${}_{2}^{+}$ A threshold. In Fig. 5(a), the black line is the absorption spectrum adapted from a synchrotron study^25^. Two series known as Worley’s third series and Ogawa-Tanaka series have been observed, labeled as WT and OT. However, some of the most prominent features in the absorption spectrum are not included in either of these two series and are labeled as UN. Figure 5b is a magnified portion of Fig. 2b from 15.72 to 16.76 eV. We can see most of the UN peaks are consistent with parallel transitions (negative for anti-alignment and positive for alignment).

For better comparison, we choose the alignment and anti-alignment delay points, labeled by the red and blue arrows in Fig. 5b. We define an asymmetry signal: ${\rm{P}}=\Delta {\rm{OD}}({\rm{Alignment}})-\Delta {\rm{OD}}({\rm{Anti}}\ {\rm{alignment}})$, shown by the black line in Fig. 5c. If we assume that the UN series have ${}^{1}{\Sigma }_{u}^{+}$ symmetry (parallel transitions), the predicted spectrum is shown by the red line. On the other hand, assuming ${}^{1}{\Pi }_{u}$ symmetry (perpendicular transitions) gives the blue line. The red line shows much better agreement with the experimental result, which suggests that these unassigned peaks have ${}^{1}{\Sigma }_{u}^{+}$ symmetry. Consider the Rydberg orbital to be $nl{\lambda }_{g/u}$, where $n$ is the principal quantum number, $l$ is the orbital angular momentum quantum number, $\lambda$ is the quantum number of the projection of orbital angular momentum onto the internuclear axis and $g/u$ means symmetric/antisymmetric orbital. Since the vibrational spacings in the UN series resemble those of the ${{\rm{N}}}_{2}^{+}{{\rm{A}}}^{2}{\Pi }_{{\rm{u}}}$ state^32,33^, these series should originate from the promotion of a $1{\pi }_{u}$ electron. So the Rydberg orbital symmetry must be $g$, which means $l$ is even^44^. Our experiment identified that the UN series are parallel transitions, so $\lambda$ should be $\pi$, which means $\lambda =1$. Since $l\ge \lambda$, $l$ must be 2, which means a $d$ orbital. We also calculated the quantum defects $\delta$ by ${\nu }_{{\rm{n}}}(v)={\nu }_{\infty }(v)-R/{(n-\delta )}^{2}$^50^, where ${\nu }_{{\rm{n}}}(v)$ is the Rydberg state with principal quantum number $n$ and in the corresponding $v$ vibrational level^33^, ${\nu }_{\infty }(v)$ is the series limit^51^; $R=109737.316\ \mathrm{c{m}}^{-1}$ is the Rydberg constant. For molecules built up from atoms of the first two rows of the periodic table, $\delta \sim 1$ for $s$ orbital, $\delta \sim 0.7$ for $p$ orbital and $\delta \sim 0$ for $d$ orbital^52^. The result $\delta \sim -0.2$ also suggests a $d$ orbital. So the transitions for these UN series are $1{\pi }_{{\rm{u}}}\to {\rm{nd}}{\pi }_{{\rm{g}}}$. Usually quantum defect calculations are not enough for the Rydberg orbital assignment, in this case, $\delta \sim -0.2$ suggests a $d$ orbital, but it can be $d{\sigma }_{g}$, $d{\pi }_{g}$, or $d{\delta }_{g}$. Sometimes, even $\delta$ value itself is difficult to get, that is why the questions about orbital assignment still exist even for simple diatomic molecules. Our assignment illustrates the value of transient absorption measurements made in the molecular frame.

For O${}_{2}$, disputed structures exist around 15 eV. The adapted absorption spectrum from^34^ is shown by the black line in Fig. 5d. Three progressions labeled as ${\rm{I}},{\rm{I}}^{\prime}$ and ${\rm{I}}^{\prime\prime}$ were observed, and converge onto the O${}_{2}^{+}{a}^{4}{\Pi }_{u}$ threshold. The Rydberg orbital assignment of these states has been the subject of considerable debate^35,36,49,50^. Similarly, Fig. 5e shows a magnified portion of Fig. 3b. Although the bound-free transition induces a parallel modulation background, we can still see that most ${\rm{I}}$ peaks fall onto the weak modulation part while most ${\rm{I}}^{\prime}$ peaks fall onto the strong modulation. The black line in Fig. 5(f) shows the asymmetry signal. By assuming ${}^{3}{\Pi }_{u}$ and ${}^{3}{\Sigma }_{u}^{-}$ symmetries for ${\rm{I}}$ and ${\rm{I}}^{\prime}$ respectively, the calculation shown by the red line in Fig. 5f achieved the best agreement. The effective quantum number of ${\rm{I}}^{\prime}$ is ${n}^{* }=2.9914$^50^, indicating the corresponding transition is $1{\pi }_{{\rm{u}}}\to 3{\rm{d}}{\pi }_{{\rm{g}}}$. The ${}^{3}{\Sigma }_{u}^{-}$ symmetry assignment for ${\rm{I}}^{\prime}$ conflicts with most previous studies. A recent photodissociation study of O${}_{2}$ around 14.6–15.2 eV also discovered some ${}^{3}{\Sigma }_{u}^{-}$ features around vibrational states $(v=4,v=5)$ of ${\rm{I}}^{\prime}$^53^. However, the most prominent features of ${\rm{I}}^{\prime}$ is $v=6$ and $v=7$^34^, which are beyond the reported spectral range of ref. ^53^. The ${\rm{I}}^{\prime\prime}$ state has much smaller absorption cross section and strongly overlaps with ${\rm{I}}$ and ${\rm{I}}^{\prime}$, which makes the symmetry identification difficult. In the future, combining more highly aligned molecular ensembles and a higher XUV spectral resolution, it shall be possible to resolve these features.

In conclusion, XUV transient absorption spectroscopy was demonstrated with aligned molecules, even though the Rydberg states have long lifetimes compared to rotational timescales^28,29^. The excitation step in the molecular frame is fast, and the dependence on molecular angle is not lost despite the complex modulation of the resulting free induction decay signal. We identified the symmetry of an unassigned series in N${}_{2}$, and found a conflict with previous assignment of the ${\rm{I}}^{\prime}$ series in O${}_{2}$. Looking forward, further control could be obtained by adding another NIR pulse after the XUV pulse, to induce dipole coupling between the excited electronic states, which can be controlled by alignment of the molecular target^54^, allowing for a clearer physical interpretation of the complex absorption spectrum. We demonstrated XUV transient absorption spectroscopy in the molecular frame using linear molecules due to their simplicity in alignment. More complex molecules can be aligned in three dimensions by using a pulse sequence^24^, permitting similar spectroscopic studies to be applied to symmetric top molecules.

## Methods

### Experimental setup

In the experiment, NIR laser pulses were generated by a Ti:sapphire laser system (100 Hz, 14 mJ, 800 nm, 50 fs). Approximately 1.6 mJ was sent into a differentially pumped hollow core fiber (250 $\upmu$m inner diameter) filled with 0.7 bar of argon to create a 10 fs pulse centered around 800 nm. This pulse was focused into a pulsed gas jet of xenon (3 bar backing pressure, 250 $\upmu$m nozzle) where high harmonic generation took place. A thin (80 $\pm$ 10 $\upmu$m) monocrystalline quartz plate was placed ~2 cm before the xenon gas jet to broaden the spectrum through self-phase modulation (SPM) and to create some second harmonic of 800 nm^55,56^. The intensity at the quartz plate was $3\times 1{0}^{13}{\rm{W}}/{{\rm{cm}}}^{2}$, below the damage threshold ($> 4\times 1{0}^{13}{\rm{W}}/{{\rm{cm}}}^{2}$) but strong enough to generate new spectral components through SPM. The bandwidth of each harmonic increased from 0.7 to 2.3 eV, the total spectrum spanning 12–24 eV. The XUV was composed of a train of about five pulses in an attosecond pulse train. A 500-$\upmu$m-diameter pinhole was used to block the residual NIR driving field and transmit most of the XUV radiation. An intensity of $2\times 1{0}^{11}{\rm{W}}/{{\rm{cm}}}^{2}$ is estimated for the residual NIR beam at the sample target based on the Stark shift (see Supplementary Note 3 and Supplementray Fig. 4). All the results in this article are reproducible when we replace the pinhole with a 100-nm-thick aluminum foil (transparent energy range: 15–73 eV) or a 350-nm-thick indium foil (transparent energy range: 11–17 eV).

A part (1 mJ) of the initial 50 fs NIR pulse was combined with the XUV pulse with a holey mirror. Both the XUV and the NIR pulses propagated noncollinearly and intersected at the absorption gas jet (250 $\upmu$m nozzle, gas density $\sim 1{0}^{18}\ {{\rm{cm}}}^{-3}$, interaction length $\sim 0.5\ {\rm{mm}}$; N${}_{2}$: $99.999 \%$ purity, 10 bar backing pressure, O${}_{2}$: $99.999 \%$ purity, 6.5 bar backing pressure) with a crossing angle of 18 mrad. The NIR pulse at the target ($3\times 1{0}^{13}\ {\rm{W}}/{{\rm{cm}}}^{2}$, pulse duration increased to 70 fs due to the dispersion of transmission optics) was used to perform impulsive alignment on the sample molecules. The transmitted XUV spectrum was dispersed in a flat-field XUV spectrometer and detected by a microchannel plate detector, with a spectral resolution of 70 meV.

## Supplementary information

Supplementary Information

## Data Availability

The data that support the findings of this study are available from the corresponding author upon reasonable request.

## Code availability

The codes used in this study are available from the corresponding author upon reasonable request.
