# Supplementary material for: Symmetry of molecular Rydberg states revealed by XUV transient absorption spectroscopy
Source: Nat Commun. 2019 Nov 21;10:5269. doi: 10.1038/s41467-019-13251-w (PMC6872753; doi:10.1038/s41467-019-13251-w)
Supplement: Supplementary file 1 — Supplementary Information [file 41467_2019_13251_MOESM1_ESM.pdf]

Supplementary Information

Symmetry of Molecular Rydberg States Revealed by XUV Transient Absorption Spectroscopy

Peng *et al.*

Supplementary Note 1: Time Response of Induced Polarization

The main text showed the time-dependent response of aligned molecules probed at different times. We approximate the N_2 molecule as a rigid rotor with a rotational constant $B_0 = 2$ cm^{-1}. With an initial rotational temperature of $T = 0$, only the $J = 0, M = 0$ rotational state is populated. We simulate the alignment process by equally populating the even J states, $J = 0, 2, 4, 6, 8, 10$, since the Raman process requires $\Delta J = \pm 2$. The degree of alignment is shown in Supplementary Fig. 1. The full revival is around $t = 8.5$ ps at which time the molecular axes are maximally parallel to the polarization axis. At $t = 8.2$ ps, the molecules are maximally perpendicular to the polarization axis (this simplified model can not reproduce 1/4 and 3/4 revivals accurately, however, it is enough for simulating full revival and is beneficial to give a physical insight to the time response of induced polarization).

The ground state wavefunction is written as

$$\left|\psi^0\right\rangle = \sum_J a_J e^{-iE_J^0 t} \left|0\right\rangle \left|JM\right\rangle , \tag{1}$$

where $E_J^0 = B_0 J(J+1)$ is the rotational energy. At a delayed time t_x, a delta-function pulse creates population in the excited electronic state,

$$\left|\psi^1\right\rangle = \sum_J b_J e^{-iE_J^1 (t-t_x)} \left|1\right\rangle \left|JM\right\rangle , \tag{2}$$

where $E_J^1 = E_1 + B_1 J(J+1)$ is the energy of the excited rotational states, including the electronic term energy E_1. The values of b_J are determined by time-dependent perturbation theory. The induced dipole moment is

$$\begin{aligned} d(t) &= \left\langle \psi^1(t) \,\middle|\, r \,\middle|\, \psi^0(t) \right\rangle + c.c. \\ &= \sum_{JJ'} a_J b_{J'}^* e^{iE_{J'}^1 (t-t_x)} e^{-iE_J^0 t} \times d_{\|} \left\langle J'M \,\middle|\, \cos\theta \,\middle|\, JM \right\rangle + c.c., \end{aligned} \tag{3}$$

where $d_{\|}$ is the electronic transition dipole moment. It is $d(t)$ that is plotted in Fig. 4 of the main text.

Fig. 4 of the main text shows the induced polarization for the case when the rotational constant of the upper and lower states are different, which is typical of almost all systems. In Supplementary Fig. 2 we show a calculation when $B_0 = B_1 = 2$ cm^{-1}. Because both the ground and excited state molecules are "rotating" at the same speed, the signal appears less chaotic. The periodicity of the ground state alignment (Supplementary Fig. 1) is more apparent. Both Supplementary Fig. 2 and Fig. 4 of the main text show the polarization signal is greatest immediately after excitation for aligned molecules, whereas for anti-aligned molecules, the signal is at first small, then increases as the molecules move into alignment 300 fs later. The overall signal is greater when the probe pulse arrives during the molecular alignment. This agrees with our experimental result: parallel transition shows stronger absorption when the XUV pulse arrives during a time of maximum alignment to the polarization direction.

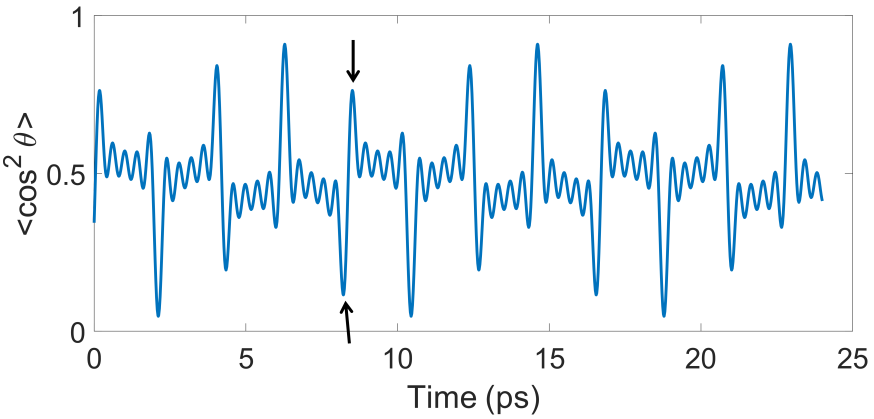

Supplementary Fig. 1. Calculated degree of alignment of the simulated N_2 molecules. The XUV probe pulse arrives when the molecules are maximally aligned parallel or perpendicular to the polarization axis, as indicated by the arrows.

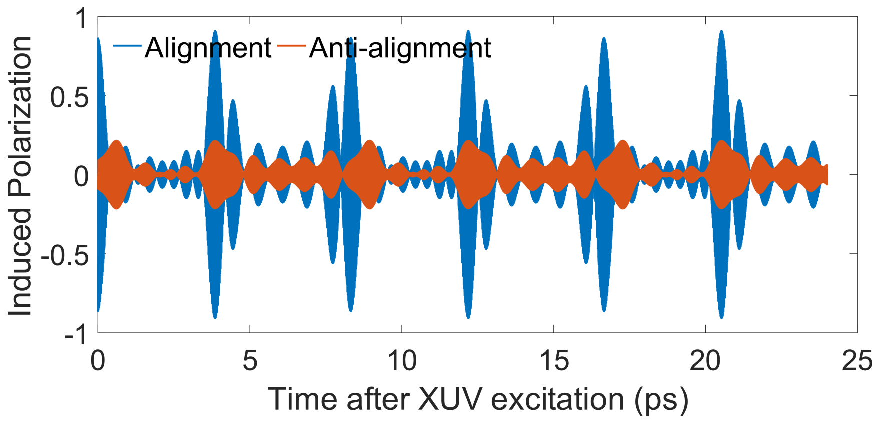

Supplementary Fig. 2. Calculated induced dipole moment for a rigid rotor linear molecule based on N_2. A rotational wavepacket is created in the ground state by the pump pulse, and the XUV pulse arrives at a later time t_x. If t_x is during a rotational revival (blue), then a greater polarization is induced. On the other hand, if t_x is when the molecules are perpendicular to the polarization, then fewer molecules are excited. The rotational constants are $B_0 = 2\ \text{cm}^{-1}$ and $B_1 = 2\ \text{cm}^{-1}$.

Supplementary Note 2: Frequency Domain Model

Molecular Alignment

When linear molecules are irradiated by a pump pulse whose duration $\tau = 70$ fs is much shorter than the rotational period, nonadiabatic field-free alignment is achieved [1, 2]. Each initial rotational eigenstate $|J_0, M_0\rangle$ will expand to a rotational wave packet $\Psi_{J_0,M_0}(t) = \sum_{J,M} a_{J,M}^{J_0,M_0}(t)|J, M\rangle$, where J is the rotational quantum number, M is the projection on the polarization axis and $a_{J,M}^{J_0,M_0}(t)$ is the amplitude for each $|J, M\rangle$ state. The time evolution of the wave packet can be calculated by solving the time-dependent Schrodinger equation. The degree of alignment is characterized by $\langle\cos^2\theta\rangle_{J_0,M_0}(t) = \langle\Psi_{J_0,M_0}(t)|\cos^2\theta|\Psi_{J_0,M_0}(t)\rangle$, in which θ is the angle of the molecular axis to the polarization axis. Finally, thermally averaged values of the degree of alignment are obtained as:

$$\langle\cos^2\theta\rangle(t) = \sum_{J_0,M_0} \rho_{J_0,M_0}(T)\langle\cos^2\theta\rangle_{J_0,M_0}(t) \tag{4}$$

where T is the rotational temperature and $\rho_{J_0,M_0}(T)$ is the Boltzmann weight function for the $|J_0, M_0\rangle$ initial state. To calculate the rotational wavepacket created by the pump pulse, we use $I = 3\times10^{13}$ W/cm^2, $\tau = 70fs$ and a rotational temperature of 100 K. The calculated $\langle\cos^2\theta\rangle(t) - 1/3$ is shown in Fig. 2(e) and Fig. 3(e) of the main manuscript.

Absorption Cross Section

The absorption cross section contains contributions from both parallel and perpendicular transitions in the molecular frame, depending on the degree of alignment:

$$\sigma(E, t) = \langle\cos^2\theta\rangle(t) \cdot \sigma_{\parallel}(E) + \left[1 - \langle\cos^2\theta\rangle(t)\right] \cdot \sigma_{\perp}(E), \tag{5}$$

where $\sigma_{\parallel}(E)$ and $\sigma_{\perp}(E)$ are absorption cross sections for parallel and perpendicular contributions, $\langle\cos^2\theta\rangle(t)$ is the alignment degree . For an isotropic medium, $\langle\cos^2\theta\rangle(t) = \frac{1}{3}$, and so $\sigma(E) = \frac{\sigma_{\parallel}(E)}{3} + \frac{2\sigma_{\perp}(E)}{3}$ [3].

Bound-Bound and Bound-Free Transitions

We consider two types of dipole-allowed transitions initiated by the XUV: bound-bound and bound-free. As N_2 and O_2 are linear molecules of $D_{\infty h}$ symmetry, with the help of the direct product table of Supplementary reference [4], all the bound-bound transitions are pure parallel or pure perpendicular. As our XUV spectrum covers several ionization thresholds of N_2 and O_2, bound-free transitions will induce a continuum absorption background. These bound-free transitions can be of dominant parallel or perpendicular character. The total absorption cross section at different alignments is defined as:

$$\begin{aligned}\sigma_{\text{total}}(E, t) = {} & \Sigma_m\langle\cos^2\theta\rangle(t) \cdot \sigma_{m\parallel}(E) + \Sigma_n\left[1 - \langle\cos^2\theta\rangle(t)\right] \cdot \sigma_{n\perp}(E) \\ & + \Sigma_c\Big[\langle\cos^2\theta\rangle(t) \cdot \sigma_{c\parallel}(E) + \left[1 - \langle\cos^2\theta\rangle(t)\right] \cdot \sigma_{c\perp}(E)\Big].\end{aligned} \tag{6}$$

The three terms in the summation correspond to bound-bound parallel, bound-bound perpendicular and bound-free transitions, respectively.

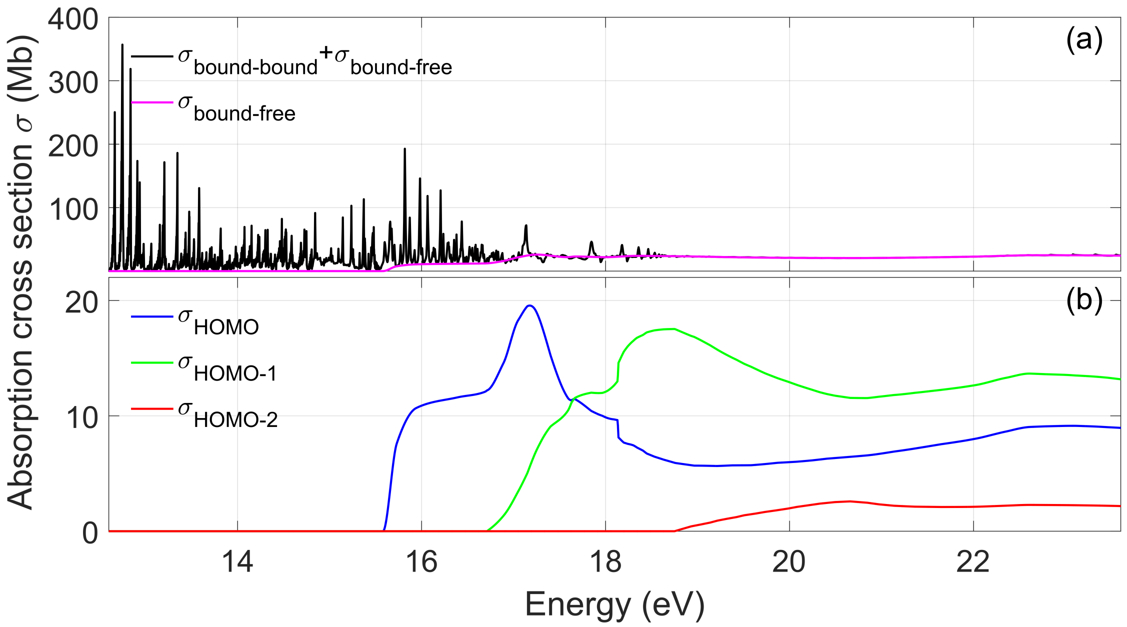

Supplementary Fig. 3. Absorption cross section of N_2. (a) High resolution absorption spectrum of N_2 adapted from [5]. Black line: total bound-bound and bound-free absorption cross section. Magenta line: total bound-free absorption cross section. (b) Partial bound-free absorption cross section.

N_2 Example

For N_2, the black line in Supplementary Fig. 3(a) shows the high resolution absorption spectrum adapted from [5]. We get the values of $\sigma_{m\|}(E)$ and $\sigma_{n\perp}(E)$ from the discrete part of the absorption cross section. Modelling the bound-free part is a bit more complicated. $\sigma_{c\|}(E)$ and $\sigma_{c\perp}(E)$ are bound-free absorption cross sections for parallel and perpendicular contribution, c can be HOMO, HOMO-1 and HOMO-2 orbitals in our situation. The continuum baseline shown by the magenta line Supplementary Fig. 3(a) gives the total bound-free absorption cross section. Combining with the ionization ratios for different orbitals [6, 7], we can get the partial bound-free absorption cross section: $\sigma_{\text{HOMO}}(E)$, $\sigma_{\text{HOMO}-1}(E)$ and $\sigma_{\text{HOMO}-2}(E)$, shown in Supplementary Fig. 3(b). But $\sigma_{c\|}(E)$ and $\sigma_{c\perp}(E)$ for each orbital are still unknown. In principle one can calculate $\sigma_{c\|}(E)$ and $\sigma_{c\perp}(E)$, but there are differences according to the computational method used [8–11]. As these bound-free absorption cross sections usually vary slowly with photon energy, we define a constant ratio $\alpha_c = \sigma_{c\|}(E)/\sigma_c(E)$. From $\sigma_c(E) = \frac{\sigma_{c\|}(E)}{3} + \frac{2\sigma_{c\perp}(E)}{3}$, we know $0 \leq \alpha_c \leq 3$, this value may vary with orbitals. Therefore, there are only three unknown constants left in Supplementary Eq. (6), which are α_{HOMO}, $\alpha_{\text{HOMO}-1}$ and $\alpha_{\text{HOMO}-2}$.

Beer-Lambert Law and Resolution Considerations

The XUV spectrum after it interacts with the macroscopic gas sample was calculated by using Beer-Lambert law:

$$I(E,t) = I_0(E)e^{-\rho\sigma_{\text{total}}(E,t)z}, \tag{7}$$

where $I_0(E)$ is the measured XUV spectrum without absorption gas shown by the black line in Fig. 1(b) of

the main manuscript, ρ is the gas density, z is the medium length, $\sigma_{\text{total}}(E,t)$ is the total absorption cross section shown in Supplementary Eq. (6). We determine the gas density by using the smooth continuum part of the measured static absorption of isotropic molecules, and infer $\rho(N_2) = 1.177 \times 10^{18}$ cm^{-3} and $\rho(O_2) = 9.77 \times 10^{17}$ cm^{-3} for a medium length of 0.5 mm. The calculated transmitted spectrum is convolved with the experimental spectral resolution to get:

$$I_c(E,t) = I(E,t) * G(E), \tag{8}$$

where $G(E) = e^{-4\ln(2)(\frac{E}{\delta E})^2}$ and $\delta E = 70$ meV.

Differential Absorption Spectrum

For isotropic sample, the total absorption cross section was given by the high resolution synchrotron data, shown by the black line in Supplementary Fig. 3(a) for N_2. Combining with Supplementary Eq. (7) and Supplementary Eq. (8), we can get the XUV spectrum after transmission through the isotropic sample $I_{\text{off}}(E)$.

For aligned sample, the total absorption cross section was calculated by Supplementary Eq. (6), and we can calculate the XUV spectrum after transmission through the aligned sample $I_{\text{on}}(E,t)$.

The predicted differential absorption spectrum is calculated in the same way as for the experimental data:

$$\Delta\text{OD}(E,t) = -\log_{10}[I_{\text{on}}(E,t)/I_{\text{off}}(E)]. \tag{9}$$

Comparing with the measured ΔOD, α_{HOMO}, $\alpha_{\text{HOMO}-1}$ and $\alpha_{\text{HOMO}-2}$ were determined by a fitting procedure. For N_2, $\alpha_{\text{HOMO}} = 2.1$, $\alpha_{\text{HOMO}-1} = 0.165$, $\alpha_{\text{HOMO}-2} = 1.6$. For O_2, $\alpha_{\text{HOMO}} = 1.3$, $\alpha_{\text{HOMO}-1} = 0.6$, $\alpha_{\text{HOMO}-2} = 2.5$. The calculation results were shown in Fig. 2(c) and Fig. 3(c) of the main manuscript. For both molecules, α values show parallel transition contribute more for HOMO and HOMO-2 orbitals while perpendicular transition contribute more for HOMO-1 orbital, which agrees with previous photonionization results [12–15].

Supplementary Note 3: Residual NIR Driving Pulse Intensity

We estimated the NIR intensity by comparing the static absorption of N_2 measured with an indium filter and with a pinhole (the NIR alignment pulse was blocked in both measurements). As shown in Supplementary Fig. 4, the absorption spectrum were normalized by the peak nearest to the ionization potential (15.58 eV). The residual NIR driving field will shift the absorption peak to higher energy due to Stark effect (the relative peak heights are also different in these two measurements, because the XUV spectrum shape will change after passing through the In filter). The shift amount is $\sim$ 12 meV, from : $U_p = e^2E^2/4mw^2$ and $I = c\varepsilon_0 E^2/2$, where U_p is the poderomotive energy, e is the electronic charge, E is the electric field, m is the mass of the electron, w is the pulse frequency, I is the pulse intensity, ε_0 is the permittivity of vacuum. The estimated residual NIR intensity is 2×10^{11} W/cm^2. The absorption linewidths in two measurements are similar which also indicate the residual NIR pulse is weak.

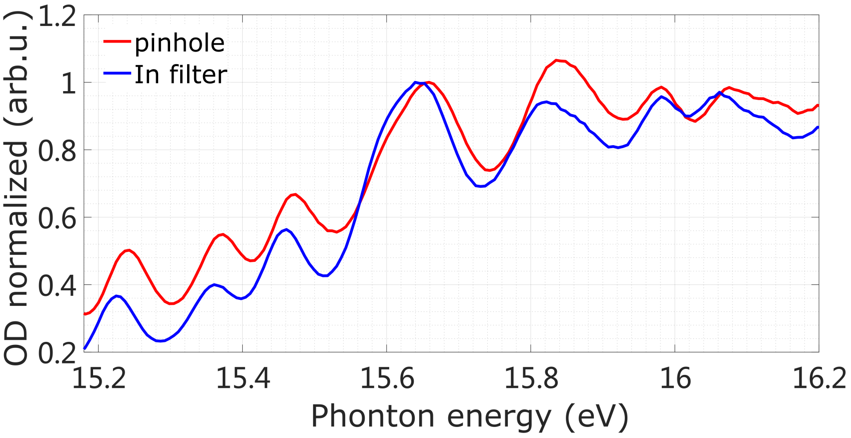

Supplementary Fig. 4. Absorption spectrum of N_2 around ionization potential. Red line: measured with a pinhole, blue line: measured with an indium filter.

Supplementary References

1. Stapelfeldt, H. & Seideman, T. Colloquium: Aligning molecules with strong laser pulses. *Rev. Mod. Phys.* **75,** 543–557 (2003).

2. Peng, P., Bai, Y., Li, N. & Liu, P. Measurement of field-free molecular alignment by balanced weak field polarization technique. *AIP Advances* **5,** 127205 (2015).

3. Cheng, Y. *et al.* Reconstruction of an excited-state molecular wave packet with attosecond transient absorption spectroscopy. *Phys. Rev. A* **94,** 023403 (2016).

4. Atkins, P. W. & Friedman, R. S. *Molecular quantum mechanics* (Oxford university press, 2011).

5. Gürtler, P., Saile, V. & Koch, E. High resolution absorption spectrum of nitrogen in the vacuum ultraviolet. *Chemical Physics Letters* **48,** 245–250 (1977).

6. Plummer, E. W., Gustafsson, T., Gudat, W. & Eastman, D. E. Partial photoionization cross sections of N_2 and CO using synchrotron radiation. *Phys. Rev. A* **15,** 2339–2355 (1977).

7. Samson, J. A., Gardner, J. & Haddad, G. Total and partial photoionization cross-sections of O_2 from 100 to 800 Å. *Journal of Electron Spectroscopy and Related Phenomena* **12,** 281–292 (1977).

8. Kosman, W. M. & Wallace, S. Complete dipole oscillator strength distribution and its moments for N_2. *The Journal of Chemical Physics* **82,** 1385–1399 (1985).

9. Semenov, S. K., Cherepkov, N. A., Fecher, G. H. & Schönhense, G. Generalization of the atomic random-phase-approximation method for diatomic molecules: N_2 photoionization cross-section calculations. *Phys. Rev. A* **61,** 032704 (2000).

10. Lucchese, R. R., Takatsuka, K. & McKoy, V. Applications of the Schwinger variational principle to electron-molecule collisions and molecular photoionization. *Physics Reports* **131,** 147–221 (1986).

11. Gerwer, A., Asaro, C., McKoy, B. V. & Langhoff, P. W. Photoexcitation and ionization in molecular oxygen: Theoretical studies of electronic transitions in the discrete and continuous spectral intervals. *The Journal of Chemical Physics* **72,** 713–727 (1980).

12. Rouzée, A. *et al.* Photoelectron kinetic and angular distributions for the ionization of aligned molecules using a HHG source. *Journal of Physics B: Atomic, Molecular and Optical Physics* **45,** 074016 (2012).

13. Rouzée, A. *et al.* Imaging the electronic structure of valence orbitals in the XUV ionization of aligned molecules. *Journal of Physics B: Atomic, Molecular and Optical Physics* **47,** 124017 (2014).

14. Marceau, C. *et al.* Molecular Frame Reconstruction Using Time-Domain Photoionization Interferometry. *Phys. Rev. Lett.* **119,** 083401 (2017).

15. Thomann, I. *et al.* Direct Measurement of the Angular Dependence of the Single-Photon Ionization of Aligned N_2 and CO_2. *The Journal of Physical Chemistry A* **112,** 9382–9386 (2008).
